# Dysregulations in hemostasis, metabolism, immune response, and angiogenesis in post-acute COVID-19 syndrome with and without postural orthostatic tachycardia syndrome: a multi-omic profiling study

**DOI:** 10.1038/s41598-023-47539-1

**Published:** 2023-11-19

**Authors:** Ali Mahdi, Allan Zhao, Emelie Fredengren, Artur Fedorowski, Frieder Braunschweig, Malin Nygren-Bonnier, Michael Runold, Judith Bruchfeld, Jannike Nickander, Qiaolin Deng, Antonio Checa, Liyew Desta, John Pernow, Marcus Ståhlberg

**Affiliations:** 1 Department of Medicine; Solna, Karolinska Institute, 171 77 Stockholm, Sweden; 2 Department of Cardiology, Karolinska University Hospital, Stockholm, Sweden; 3 Department of Physiology and Pharmacology, Karolinska Institutet, Stockholm, Sweden; 4 Division of Physiotherapy, Department of Neurobiology, Care Sciences and Society, Karolinska Institutet, Stockholm, Sweden; 5 Women’s Health and Allied Health Professionals Theme, Medical Unit Occupational Therapy and Physiotherapy, Karolinska University Hospital, Stockholm, Sweden; 6 Department of Respiratory Medicine and Allergy, Karolinska University Hospital, Stockholm, Sweden; 7 Department of Infectious Diseases, Karolinska University Hospital, Stockholm, Sweden; 8 Division of Infectious Diseases, Department of Medicine Solna, Karolinska Institutet, Stockholm, Sweden; 9 Department of Clinical Physiology, Karolinska University Hospital, and Karolinska Institutet, Stockholm, Sweden; 10 Unit of Integrative Metabolomics, Institute of Environmental Medicine, Karolinska Institute, Stockholm, Sweden

**Keywords:** Arrhythmias, Molecular medicine

## Abstract

Post-acute COVID-19 (PACS) are associated with cardiovascular dysfunction, especially postural orthostatic tachycardia syndrome (POTS). Patients with PACS, both in the absence or presence of POTS, exhibit a wide range of persisting symptoms long after the acute infection. Some of these symptoms may stem from alterations in cardiovascular homeostasis, but the exact mechanisms are poorly understood. The aim of this study was to provide a broad molecular characterization of patients with PACS with (PACS + POTS) and without (PACS-POTS) POTS compared to healthy subjects, including a broad proteomic characterization with a focus on plasma cardiometabolic proteins, quantification of cytokines/chemokines and determination of plasma sphingolipid levels. Twenty-one healthy subjects without a prior COVID-19 infection (mean age 43 years, 95% females), 20 non-hospitalized patients with PACS + POTS (mean age 39 years, 95% females) and 22 non-hospitalized patients with PACS-POTS (mean age 44 years, 100% females) were studied. PACS patients were non-hospitalized and recruited ≈18 months after the acute infection. Cardiometabolic proteomic analyses revealed a dysregulation of ≈200 out of 700 analyzed proteins in both PACS groups vs. healthy subjects with the majority (> 90%) being upregulated. There was a large overlap (> 90%) with no major differences between the PACS groups. Gene ontology enrichment analysis revealed alterations in hemostasis/coagulation, metabolism, immune responses, and angiogenesis in PACS vs. healthy controls. Furthermore, 11 out of 33 cytokines/chemokines were significantly upregulated both in PACS + POTS and PACS-POTS vs. healthy controls and none of the cytokines were downregulated. There were no differences in between the PACS groups in the cytokine levels. Lastly, 16 and 19 out of 88 sphingolipids were significantly dysregulated in PACS + POTS and PACS-POTS, respectively, compared to controls with no differences between the groups. Collectively, these observations suggest a clear and distinct dysregulation in the proteome, cytokines/chemokines, and sphingolipid levels in PACS patients compared to healthy subjects without any clear signature associated with POTS. This enhances our understanding and might pave the way for future experimental and clinical investigations to elucidate and/or target resolution of inflammation and micro-clots and restore the hemostasis and immunity in PACS.

## Introduction

The SARS-CoV-2 virus causes COVID-19 which has affected hundreds of millions of people worldwide since it first emerged in late 2019. While most infected individuals recover fully within a few weeks, some experience symptoms that persist for months after acute infection or develop symptoms after a short period of recovery. This novel condition is referred to as post-acute COVID-19 syndrome (PACS)^1^ and is defined as persisting symptoms > 12 weeks after contracting an acute SARS-CoV-2 infection^2^. It is estimated that the prevalence of PACS is approximately 10% among non-hospitalized patients with a mild acute infection^3,4^. PACS is a complex and multi-faceted condition that can affect various organ systems, including the respiratory, cardiovascular, neurological and musculoskeletal systems^1^. Some of the most common symptoms of PACS include fatigue, shortness of breath, chest pain, joint and muscle pain, palpitations and cognitive impairment. However, the pathophysiology underlying PACS is to this date incompletely understood and needs further investigation. We were among the first to report that PACS can be associated with postural orthostatic tachycardia syndrome (POTS)^5^ and have highlighted the importance of cardiovascular dysautonomia in this patient population^2^. A recent study estimates the prevalence of POTS to be 30% in severely affected PACS patients referred to a tertiary specialist center^6^. More recently we have shown that POTS associated with PACS is accompanied with microvascular endothelial dysfunction which translates into reduced endothelial dependent cardiac stress perfusion^7^.

The pathophysiological mechanisms underlying PACS remain unknown but proposed causes are persisting SARS-CoV-2 virus, immune system dysregulation, autoimmunity, endothelial cell inflammation (endotheliitis) associated with micro clot formation or neurological dysregulation^8^. The clinical and pathophysiological association between PACS and POTS also remain elusive. While the prevalence of POTS in PACS has been reported in case series^9^, detailed studies exploring pathological mechanisms underlying PACS-related POTS are lacking. Possible overlap, but also distinctions among various PACS phenotypes, including POTS and other forms of cardiovascular complications, might exist. Thus, additional knowledge regarding alterations in key signaling pathways is needed to improve our understanding of this complex condition.

Therefore, we sought to provide a foundation for future mechanistic studies by applying an unbiased, multi-omic approach analyzing cardiometabolic plasma proteins, proinflammatory cytokines/chemokines and sphingolipids in patients with PACS with and without POTS compared to healthy controls. Our hypothesis posits significant molecular changes in PACS, particularly in cases associated with POTS, aiming to lay a foundation for future mechanistic studies and enhance our understanding of these intricate conditions.

## Material and methods

### Study population

Forty-two non-hospitalized PACS patients were recruited from the tertiary post-acute COVID-19 clinic at Karolinska University Hospital, Stockholm, Sweden. All subjects were diagnosed with PACS by a multidisciplinary team in accordance with the current WHO definition^10^ and underwent a head-up TILT test due to clinical suspicion of POTS^11^. All patients were referred to the multidisciplinary clinic due to prolonged and disabling symptoms with at least 50% sick-leave for at least six months after the acute COVID-19 illness. Subjects were stratified according to the presence (n = 20) (PACS + POTS) or absence (n = 22) (PACS-POTS) of POTS defined as a persistent rise in heart rate of > 30 beats/min or a heart rate > 120 beats/min within 10 min of tilt-up test in line with current diagnostic guideline criteria for POTS^12^. The subjects were recruited consecutively with the aim of recruiting 50% with and 50% without POTS. This was an exploratory study based on a patient series collected at tertiary center and age and sex matched controls in 2:1 ratio as usually applied in unbiased exploratory studies. As part of the clinical evaluation, all subjects underwent an ambulatory 24-h Holter electrocardiogram and mean frequency over 24 h was collected. Information on comorbidities and medication at the time of inclusion was collected from the patient charts. Age and sex –matched healthy subjects without history of cardiovascular disease, diabetes, autoimmune disease, cancer, or immunosuppressive treatment served as controls (n = 21). Participants were informed about the purpose and possible risks of the study, and all gave their informed consent. The investigation was approved by the Swedish Ethical Review Authority in Stockholm and conducted according to the Declaration of Helsinki.

To assess the symptom burden, PACS patients were asked to fill in a questionnaire of their current symptoms on the day of blood sampling. To simplify the assessment of symptom burden for the PACS patients, the questionnaire covered the most common symptoms in PACS (26 symptoms) based on our clinical experience and one of the few reports at the time of the study initiation^13^. The subjects were asked to answer in a yes/no fashion. Furthermore, patients were asked to fill in Malmö POTS Symptom (MAPS) score questionnaire at the clinical consultation in the post-acute COVID-19 multidisciplinary clinic. This symptom score covers the twelve most common symptoms and their severity in POTS and has been validated before the pandemic^14^.

Subjects were asked to refrain from all medications on the day of sampling and fast 12 h prior to their visit. Supine whole blood was collected in EDTA tubes and centrifuged at 1000×*g* for 10 min. Aliquots of plasma were stored at – 80 until the analysis.

### Plasma protein and cytokine profiling

**Figure 1:**
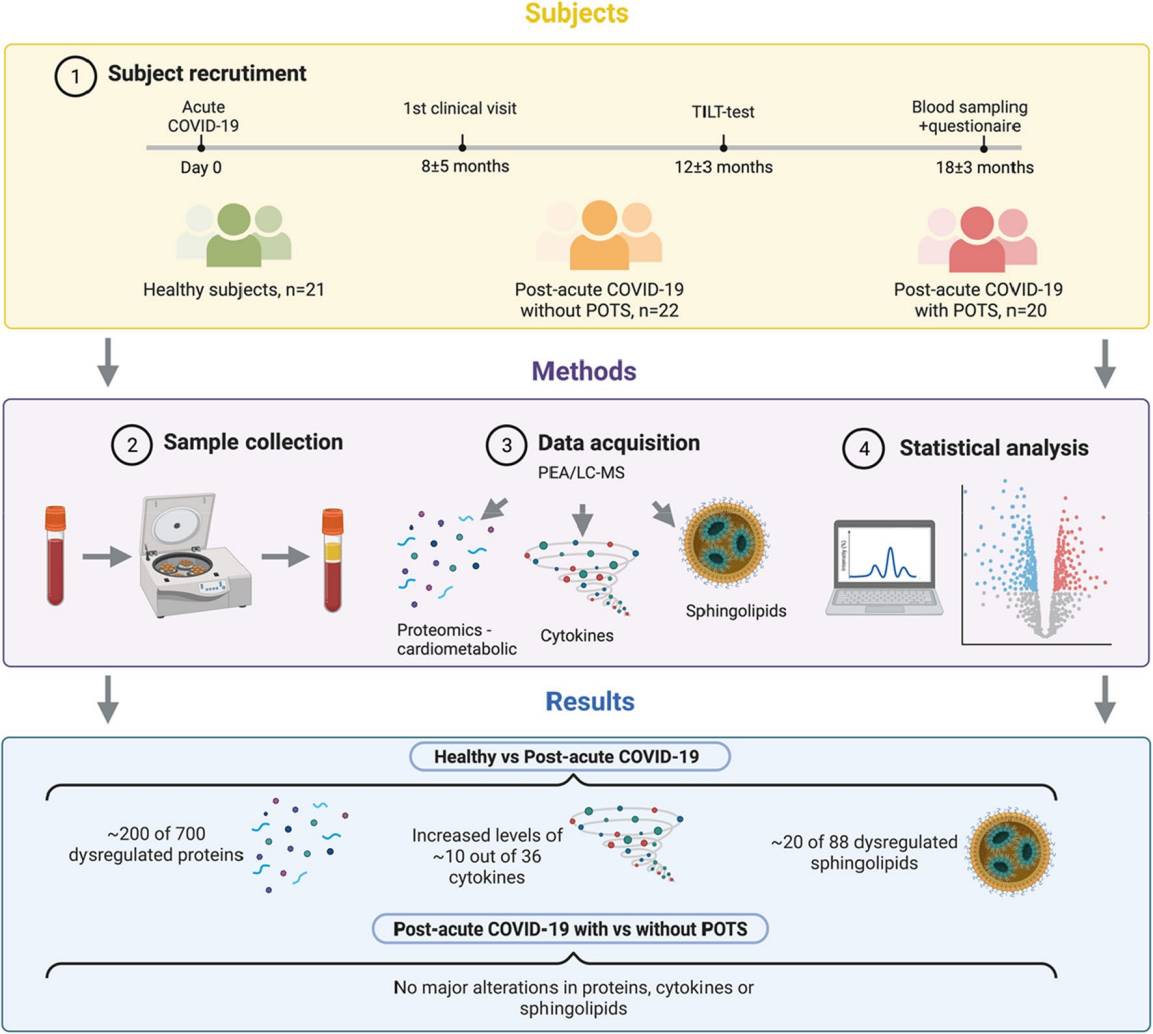
Overview of the workflow and key findings. Patients with post-acute sequelae of COVID-19 (PACS) without and with postural orthostatic tachycardia syndrome (POTS) were recruited from which peripheral blood plasma was collected and analyzed with a proteomics platform Olink, cardiometabolic I and II, covering 700 proteins, 36 cytokines and 88 sphingolipids. Detailed analyses show that a large proportion of the analyzed proteins, cytokines and sphingolipid were dysregulated in PACS irrespective of the presence of POTS. Few or no alterations were detected in the PACS group with compared to without POTS.

The overall study design is presented in Fig. 1. Plasma samples were analyzed using a multiplex proximity extension assay (OLINK, Uppsala, Sweden)^15^. Briefly, plasma proteins are recognized by pairs of oligonucleotide-coupled antibodies, and after recognition and binding, the paired antibodies will form overlapping sequences with their bound oligonucleotides. This overlap is then a PCR-target that can be quantified using real-time PCR. In total, 729 cardiometabolic proteins were analyzed using two OLINK panels (Cardiometabolic-I and Cardiometabolic-II) and cytokines were analyzed using the Target 48 Cytokine panel, including 45 different cytokines and chemokines. The proteins were analyzed in one batch, eliminating batch-to-batch variability and samples were randomly assigned to different wells on the plates, to allow for intensity normalization and reduce the technical variability. Twenty-nine proteins fell below the cut-off for LOD (these proteins are listed in Supplementary File 1) which resulted in 700 remaining proteins for analysis. Nine of the included cytokines/chemokines in the predefined panel were below the LOD (these cytokines/chemokines are listed in Supplementary file 2) which resulted in 36 remaining cytokines for analysis.

### Sample preparation for sphingolipid profiling

To quantify sphingolipids in plasma, samples were divided into two extraction batches each one containing samples, 3 quality controls of extraction (QCExt) and 1 blank of extraction (BLExt). For each batch, samples were first thawed at 4 °C in the fridge and vortexed for 20 s. Afterwards, a volume of 25 µl of each sample was aliquoted on an Eppendorf tube. Then, 10 µl of the internal standard mixture, containing deuterium labelled sphingolipids (at least one per class) was added to each sample. Then, a volume of 250 µl of methanol was added to each sample. Eppendorf tubes were then closed and vortexed for 10 s. Afterwards, samples were sonicated for 15 min on an ice bath to avoid the temperature increasing above 20 °C. Samples were then centrifuged at 10,000 g for 15 min. An aliquot of 120 µl was finally transferred to an LC–MS vial equipped with a 150 µl insert. All samples and QCExts were analyzed within one day of extraction.

### LC–MS/MS sphingolipid profiling

Chromatographic separation was carried out on an ACQUITY UPLC System with a sample manager cooled to 8 °C (both from Waters Corporation, Milford, MA, USA). Sphingolipids were separated on a Zorbax Rapid Resolution RRHD C18 Column, 80 Å, 1.8 µm, 2.1 mm × 100 mm (Agilent Technologies; Product Number: 758700-902) using a guard column (Agilent Technologies, PN: 821725-901) (5 × 2 mm, 1.8 μm particle size). Mobiles phases A and B consisted of 5 mM ammonium formate (Sigma; PN: 70221)/0.2% formic acid (Optima, Fisher-Scientific, PN: 10596814) in water and in methanol (Optima, Fisher Scientific, PN: A456-212), respectively. Separation was carried out at a 450 μl/min flowrate and a column temperature was held at 40°C. The following chromatographic gradient was used: 0 min, 75% B; time range 0 → 1 min, 75% B (constant); time range 1 → 5 min, 85 → 100% B (linear increase); time range 5 to 15.2 min, 100% B (isocratic range); time range 15.2 → 15.3 min, 100 → 75% B (linear decrease); time range 15.3 → 16 min, 75% B (isocratic column conditioning). Samples were then analysed on a Waters Xevo® TQ-S system equipped with an Electrospray Ion Source (ESI) and ScanWave™ collision cell technology operating in the positive mode. A class specific single reaction monitoring (SRM) transition for each sphingolipid and internal standard was used. For sphingolipids with a standard available, an injection of the standard was used to assess the retention time for the selected SRM. For sphingolipids without a standard available, retention time was predicted based on their number of carbons and unsaturations relative to compounds calibrated with a standard. Specific information on SRM, retention times and compound information are detailed in Supplementary File 4. The method does not distinguish glycosylated species (GlcCer) from galactosylated species (GalCer), and Glc sphingolipids are therefore potentially a mixture of the two species.

### Statistical analyses

Analysis of protein, cytokine and lipid data was performed in R (version 4.2.2 R Foundation for Statistical Computing, Vienna, Austria; http://www.r-project.org). Packages dplyr (version 1.1.0) and tidyr (version 1.3.0) were used for data preprocessing. The package clusterProfiler (version 4.6.0) was used for enrichment analysis. Packages ggplot2 (version 3.4.1), EnhancedVolcano (version 1.16.0), FactoMineR (version 2.7) and pheatmap (version 1.0.12) were used for data visualization.

### Proteomic and cytokine analysis

For the plasma protein profiling, the abundance of each protein was recalculated to a relative log2-scale in arbitrary units. For the plasma cytokine profiling, each cytokine was calculated to a specific concentration. Cytokines/chemokines were log-transformed before subsequent downstream processing. Proteins or cytokines/chemokines that were detected in less than 25% of samples were discarded, and any missing values were set to the limit of detection (LOD). After preprocessing the data, differentially expressed proteins and cytokines were obtained using the limma package^16^ and proteins were considered significantly differentially expressed if they had a Benjamini–Hochberg adjusted p-value < 0.05.

### Lipidomic analysis

For the sphingolipid profiling, values were normalized regarding concentration or relative abundance against internal controls. The values were log-transformed before downstream analysis. For differential expression, the limma package was used, and lipids were considered significantly differentially expressed if they had a Benjamini–Hochberg adjusted p-value < 0.05.

## Results

### Subjects

**Figure 2:**
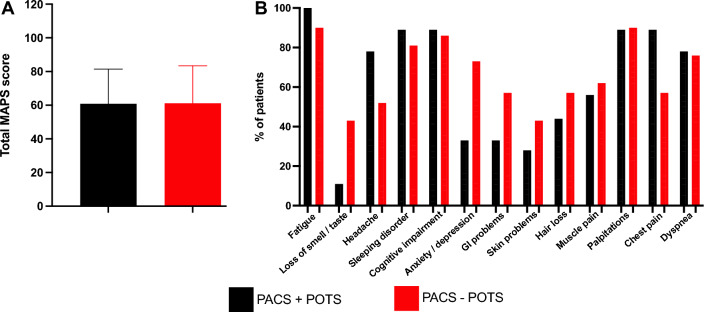
No significant differences in a semiquantitative assessment of symptom burden with the Malmö POTS (MAPS) score (0–10 per item, 12 items) in PACS with, compared to without, POTS (**A**). Similar proportion of PACS patients with, compared to without POTS, reporting common symptoms in PACS, with some exceptions, as listed (**B**).

Study sample characteristics and symptom burden are shown in Table 1 and Fig. 2. As depicted in Table 1, most of the subjects were females. The mean age was comparable between the groups and did not differ significantly. The PACS patients were free from comorbidities except for a subset of PACS-POTS (40%) who declared depression/anxiety. For this reason, a larger proportion of patients in this group were on sedative and hypnotics. The majority (60%) of patients in the PACS + POTS group were on ivabradine. As expected, the increase in heart rate following head-up tilt test was higher in the PACS + POTS group. However, the mean heart rate over 24 h did not differ between the two PACS groups. The symptom burden between the two PACS groups did not differ in major aspects as MAPS score was similar (Fig. 2A) and the proportion of patients reporting common symptoms (13 of 26 most common are shown) in PACS at the day of sampling was, overall, comparable with some exceptions (Fig. 2B).

**Table 1:** Baseline characteristics of the study population included in the current study.

Variable	Healthy controls, n = 21	PACS, n = 42	PACS + POTS, n = 20	PACS–POTS, n = 22	p-value, PACS vs healthy controls	p-value, POTS + vs POTS –
Demographics						
Female sex, n (%)	20 (95)	41 (98)	19 (95)	22 (100)	> 0.99	> 0.99
Age, years	43 ± 6	42 ± 10	39 ± 11	44 ± 11	0.52	0.09
PACS-duration (months)	N/A	18 ± 3	18 ± 3	19 ± 3	N/A	0.36
Preexisting comorbidities before PACS diagnosis, n (%)						
Hypertension	0 (0)	2 (5)	1 (5)	1 (5)	> 0.99	> 0.99
Diabetes mellitus	0 (0)	0 (0)	0 (0)	0 (0)	> 0.99	> 0.99
Asthma	0(0)	7 (17)	4 (20)	3 (14)	0.08	0.69
Depression/anxiety/clinical burnout	0 (0)	11 (26)	2 (10)	9 (40)	0.01	0.04
ME/CFS	0 (0)	1 (2)	0(0)	1 (5)	> 0.99	> 0.99
EDS	0 (0)	3 (7)	2 (10)	1 (5)	0.54	0.60
Hypothyroidism	0 (0)	3 (7)	2 (10)	1 (5)	0.54	0.60
Pharmacological treatment at inclusion, n (%)						
Ivabradine	0 (0)	16 (38)	12 (60)	4 (18)	0.001	0.01
β-blockers	0 (0)	13 (31)	6 (30)	7 (32)	0.002	> 0.99
Ca^2+^ channel blockers	0 (0)	3 (7)	2 (10)	1 (5)	0.003	0.61
SSRI	0 (0)	10 (24)	5 (25)	5 (23)	0.02	> 0.99
Inhalation steroids	0 (0)	10 (24)	4 (20)	6 (27)	0.02	0.72
Sedative and hypnotics	0 (0)	16 (38)	5 (25)	11 (50)	0.01	0.12
POTS-associated						
Delta HR on HUT (bpm)	N/A	N/A	40 ± 15	19 ± 8	N/A	< 0.001
Average HR on 24 h Holter ECG	N/A	N/A	77 ± 7	75 ± 9	N/A	0.27

Data are presented as mean and standard deviation or numbers (n) and %. Significant differences were calculated with student’s t-test or chi-square test and p-values are shown.

*b-blocker* beta receptor blockers, *bpm* beats per minute, *ECG* electrocardiogram, *EDS* Ehler-Danlos syndrome, *HR* heart rate, *HUT* head-up TILT test, *ME/CFS* Myalgic encephalomyelitis/chronic fatigue syndrome, *N/A* not applicable, *PACS* post-acute COVID-19 syndrome, *POTS* postural orthostatic tachycardia syndrome, *SSRI* selective serotonin reuptake inhibitor.

### Plasma proteomic profiling

**Figure 3:**
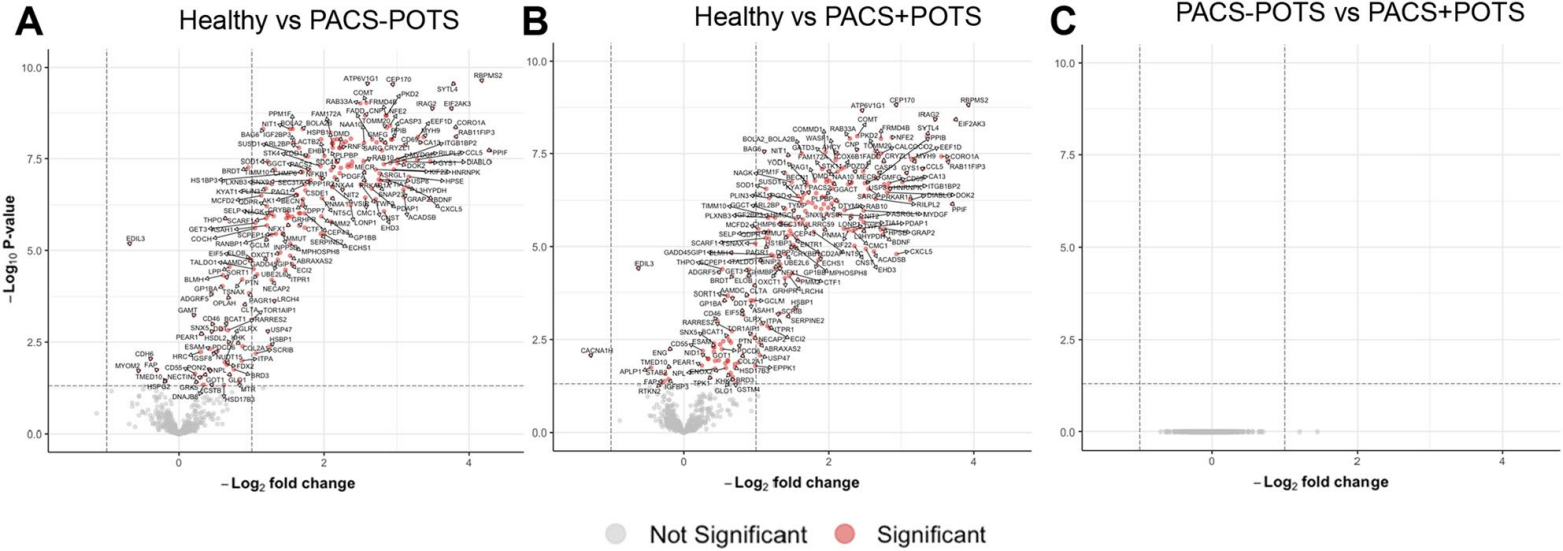
Volcano plots for 700 proteins are shown as individual dots, for comparisons healthy controls vs PACS + POTS patients (**A**), healthy controls vs PACS-POTS patients (**B**) and PACS-POTS vs PACS + POTS patients (**C**). Non-significant (grey) and significant (red) changes are shown. Adjusted p < 0.05 was considered statistically significant.

**Figure 4:**
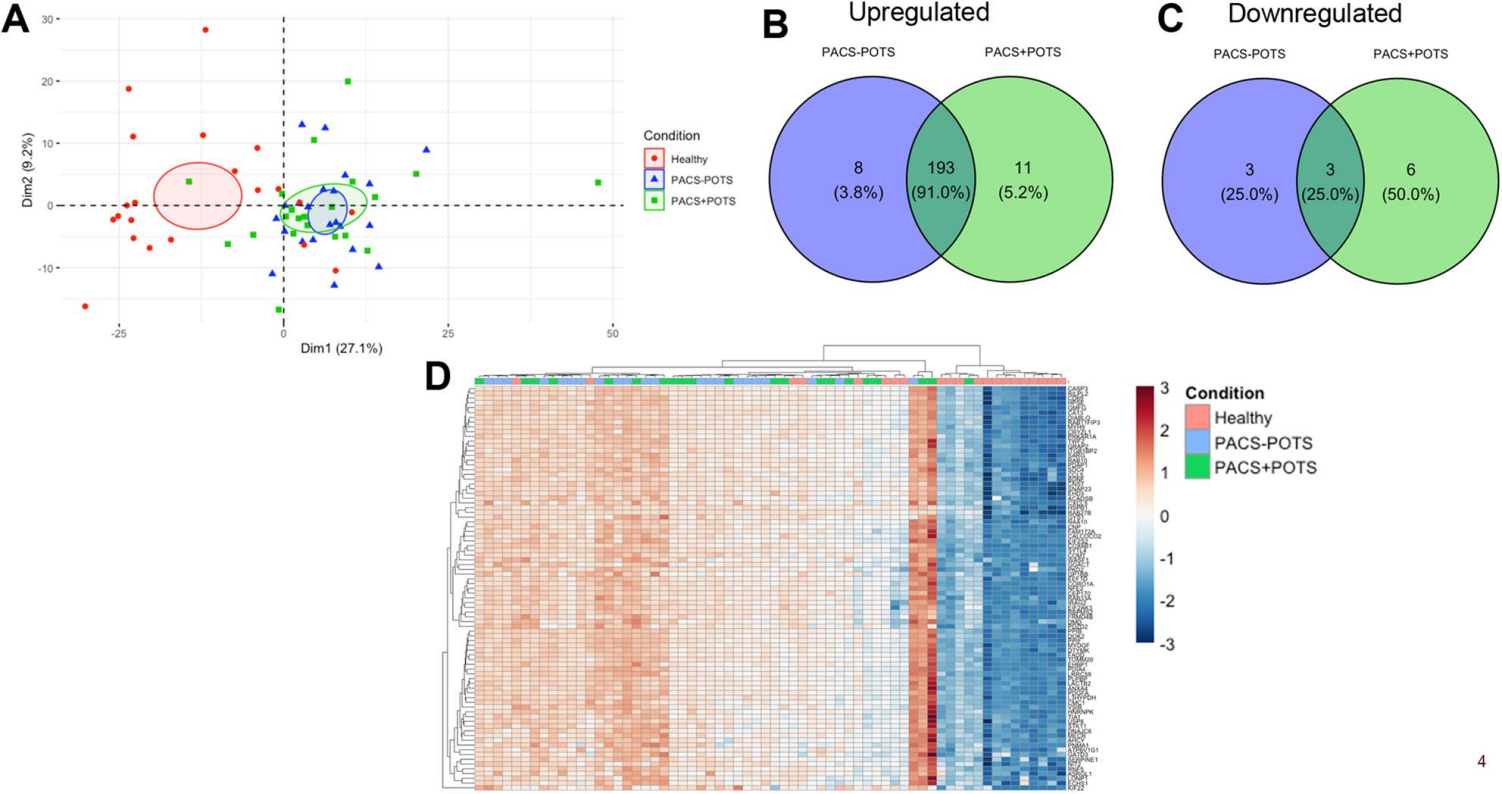
Principal component analysis (PCA) for dysregulated proteins (**A**). Plot colored by sample group. Venn diagrams of differentially upregulated (**B**) and downregulated (**C**) plasma proteins for PACS + POTS and PACS-POTS patients compared to healthy controls. Interactive heatmap with unsupervised hierarchical clustering of samples based on proteins with a log2 fold change > 2 (**D**).

In the differential expression analysis, we found 204 proteins upregulated in the PACS + POTS group when compared to healthy controls (Fig. 3A), and 201 proteins upregulated in the PACS-POTS group (Fig. 3B). As POTS has been described as a distinct phenotype of PACS, we assessed differences in the proteome between the two PACS groups. Interestingly, there were no significantly altered proteins between the groups, and there were also few proteins with a log2-fold change > 1 (Fig. 3C). Next, we performed principal component analysis (PCA) of our proteomics dataset, which showed clear distinction between healthy controls and both PACS groups, but no major distinction between the two PACS groups (Fig. 4A). Among the upregulated proteins in both PACS groups vs. healthy controls, we observed more than 90% overlap between the PACS groups, with only a few proteins uniquely upregulated in each of the groups (Fig. 4B). Most investigated proteins were upregulated, while only 9 proteins in the PACS + POTS group and 6 in the PACS-POTS group were downregulated (Fig. 4C). All proteins with associated log2-fold changes, raw p-values and adjusted p-values are listed in Supplementary File 1.

Finally, an interactive heatmap with unsupervised hierarchical clustering for the significantly different proteins with log2-fold change >  ± 2 was used to further depict the differentially expressed proteins in healthy controls and PACS patients. This resulted in a clear cluster of healthy controls, but no major clusters comparing PACS + POTS with PACS-POTS, further supporting the similarities in the plasma proteome of PACS patients regardless of concomitant POTS (Fig. 4D).

To further dissect the dysregulated outcomes across the groups and to find possible small differences that were undetected due to lack of power, we chose to look whether there were any uniquely up- or downregulated proteins when comparing PACS + POTS and PACS-POTS groups, respectively, with healthy controls. This analysis showed only minor differences between the PACS + POTS and PACS-POTS groups, as there were 11 and 8 uniquely upregulated proteins for the PACS + POTS and PACS-POTS groups, respectively, as well as 6 and 3 uniquely downregulated proteins for the PACS + POTS and PACS-POTS groups, respectively (Fig. 4B,C). These proteins are listed in Supplementary File 1. Taken together, PACS patients had a clearly dysregulated plasma proteome with a substantial proportion (almost 30%) of dysregulated proteins compared to healthy controls, but there were no major differences between PACS patients with or without POTS.

### Protein functionality analysis

**Figure 5:**
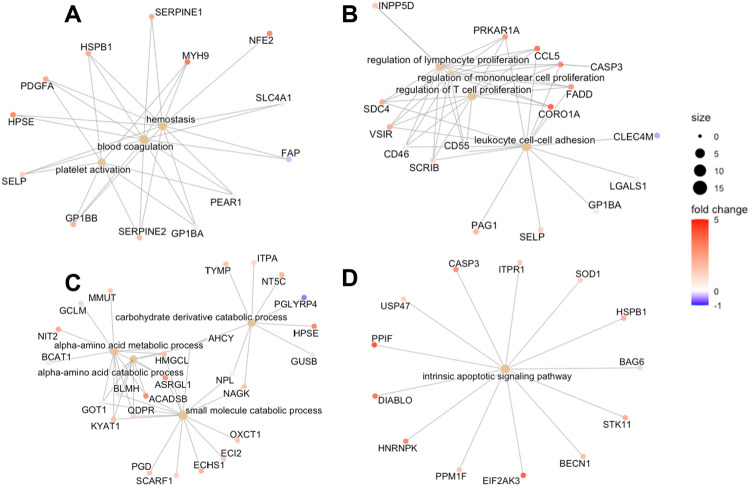
Gene ontology enrichment analysis of dysregulated proteins displayed as network plots in all PACS patients (with and without POTS) vs healthy controls. Terms related to blood clotting (**A**), Inflammation and the immune system-related signaling (**B**), Terms related to metabolism (**C**), and Apoptosis-related signaling (**D**).

We next sought to gain knowledge about the potential function of the plasma protein dysregulation seen in PACS. As there was no difference between the PACS + POTS and PACS-POTS groups, we combined the data from these groups and analyzed them vs. healthy controls. Gene ontology (GO) pathway enrichment analysis led to a large amount of significantly altered pathways, including processes involved in hemostasis, inflammation, amino acid metabolism and apoptosis (Fig. 5A–D). Network plots were then used to depict which specific proteins were altered in each pathway. Interestingly, for hemostasis and coagulation, we found that key proteins such as SERPINE1 (also known as plasminogen activator inhibitor 1) were strongly upregulated in PACS patients, implying increases in clotting susceptibility (Fig. 5A). Moreover, CCL5, a chemokine often expressed by T-cells involved in inflammation, was upregulated in PACS patients (Fig. 5B), implying a key role of T-cells in the hyperinflammatory response often seen in PACS patients. Finally, amino acid metabolism seemed to be increased with expansion of key amino acid metabolic enzymes (Fig. 5C), whilst key apoptotic markers such as Caspase 3 and DIABLO were upregulated in PACS patients (Fig. 5D), indicating activation of apoptotic processes.

### Plasma cytokine profiling

**Figure 6:**
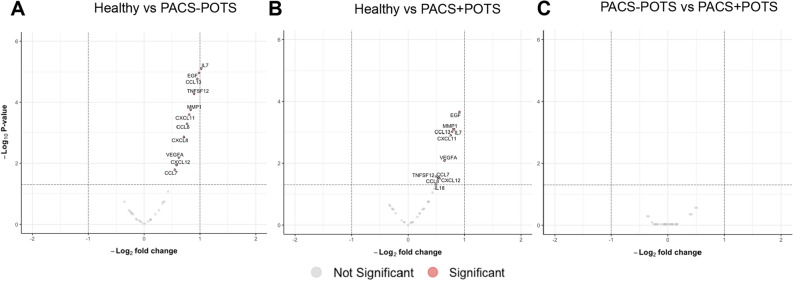
Volcano plots for 36 analyzed cytokines, with individual cytokines shown as individual dots, for comparisons healthy controls versus PACS + POTS patients (**A**), healthy controls versus PACS-POTS patients (**B**) and PACS-POTS versus PACS + POTS patients (**C**). Non-significant (grey) and significant (red) changes are shown. Adjusted p < 0.05 was considered statistically significant.

**Figure 7:**
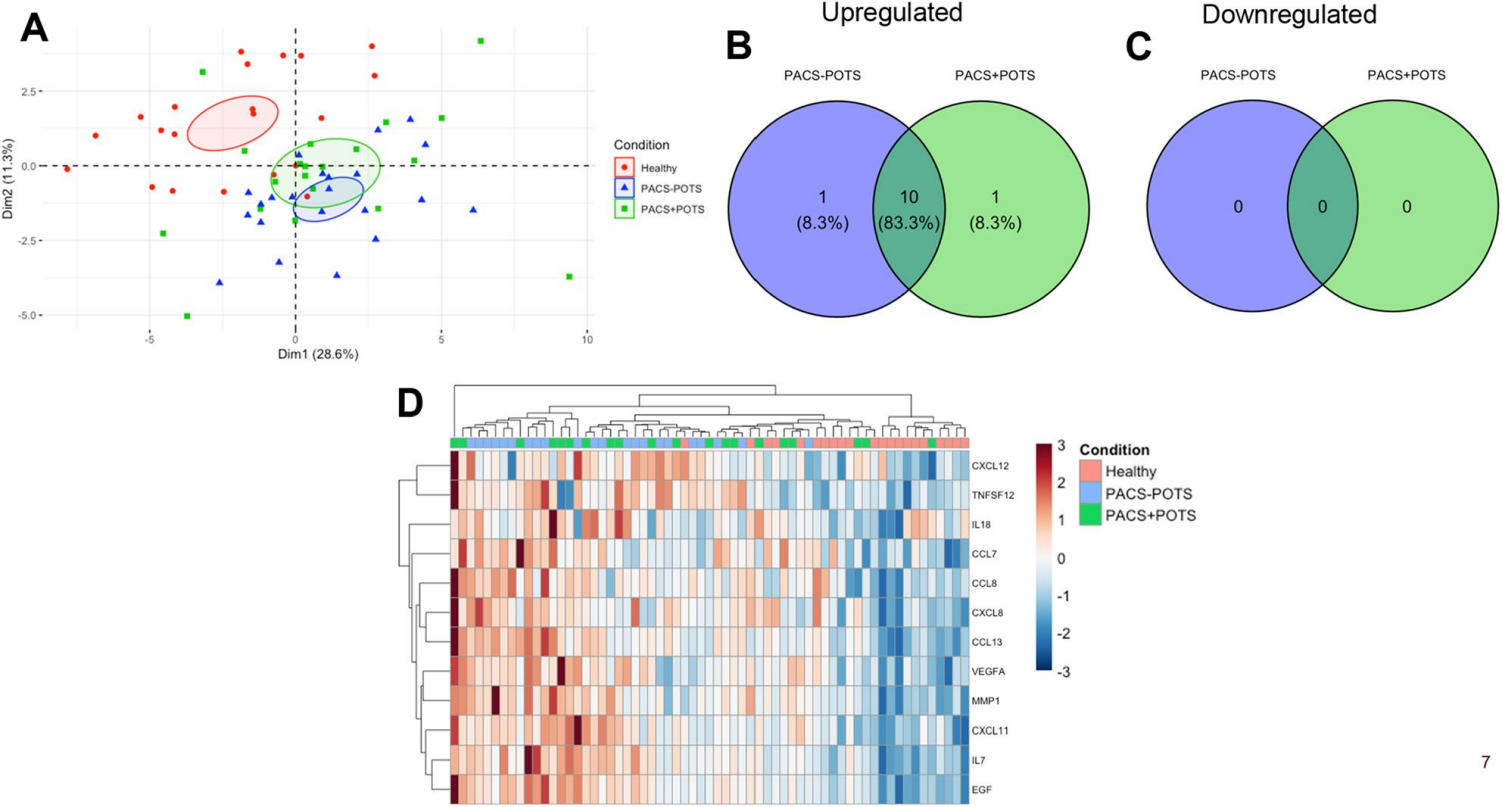
Principal component analysis (PCA) for cytokines (**A**). Plot colored by sample group. Venn diagrams of differentially upregulated (**B**) and downregulated (**C**) cytokines for PACS + POTS and PACS-POTS patients when compared to healthy controls. Interactive heatmap with unsupervised hierarchical clustering of samples based on an adjusted p-value < 0.05 (**D**).

We next analyzed cytokine profiles since both acute COVID-19 and PACS are associated with hyperinflammation, and our proteomics data suggested dysregulation in T-cell responses (Fig. 5B). Differential expression analysis showed similar patterns in cytokine dysregulation in both PACS groups compared to healthy controls, but no significant difference between PACS + POTS and PACS-POTS (Fig. 6A–C). Like the proteomics data, initial PCA analysis displayed clear difference between healthy controls and both PACS groups, but no major difference between PACS + POTS and PACS-POTS (Fig. 7A). There was a large overlap in the upregulated cytokines in both PACS groups vs healthy controls (Fig. 7B), with only one uniquely upregulated cytokine in each of the groups (Supplementary File 2). However, there were no downregulated cytokines in any of the groups (Fig. 7C), further strengthening the distinct pro-inflammatory signature of the PACS population. Interestingly, several of the commonly upregulated cytokines such as vascular endothelial growth factor-A (VEGF-A) and epidermal growth factor (EGF) play a role in angiogenesis. Unsupervised hierarchical clustering reaffirmed the results of the differential expression analysis by showing clear clustering when healthy controls were compared with PACS, but no pronounced clustering when PACS + POTS were compared with PACS-POTS (Fig. 7D). All cytokines with associated log2 fold changes, raw p-values and adjusted p-values are listed in Supplementary File 2.

### Plasma sphingolipid profiling

It has previously been shown that sphingolipids, in particular ceramides, represent potential drivers of cardiovascular disease through various actions, including accumulation in tissues relevant for cardiovascular disease such as the vasculature and the heart with adverse metabolic regulation^17^. A proposed key-mechanism by which sphingolipids act as mediators of cardiovascular disease is through their pleiotropic effect as inhibitors of nitric oxide formation and increased production of reactive oxygen species^17,18^. Since our previous work showed that PACS + POTS patients frequently presented with microvascular dysfunction (MVD), we hypothesized that sphingolipid-related dysregulation could accompany POTS and/or MVD in PACS patients. Using a targeted LC–MS based lipidomic approach specifically for sphingolipid detection, we analyzed a total of 88 sphingolipids in our cohort.

**Figure 8:**
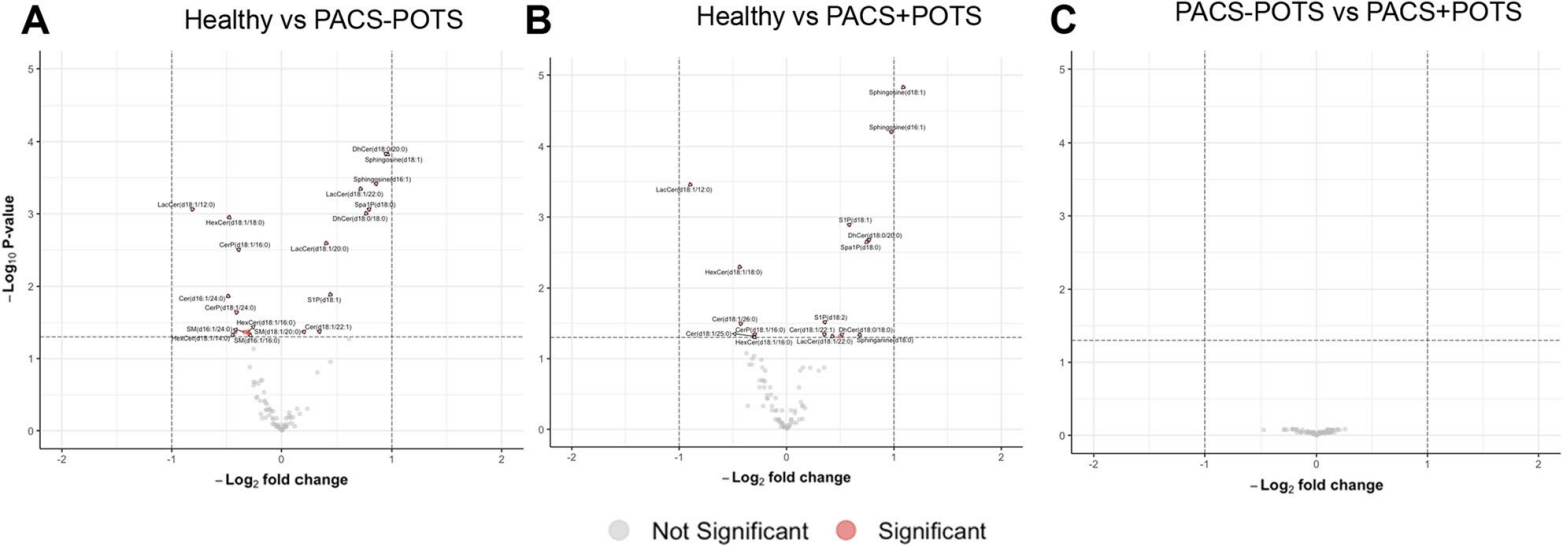
Volcano plots for 88 different sphingolipids, with individual sphingolipids shown as individual dots, for comparisons healthy controls versus PACS + POTS patients (**A**), healthy controls versus PACS-POTS patients (**B**) and PACS-POTS versus PACS + POTS patients (**C**). Non-significant (grey) and significant (red) changes are shown. Adjusted p < 0.05 was considered statistically significant.

**Figure 9:**
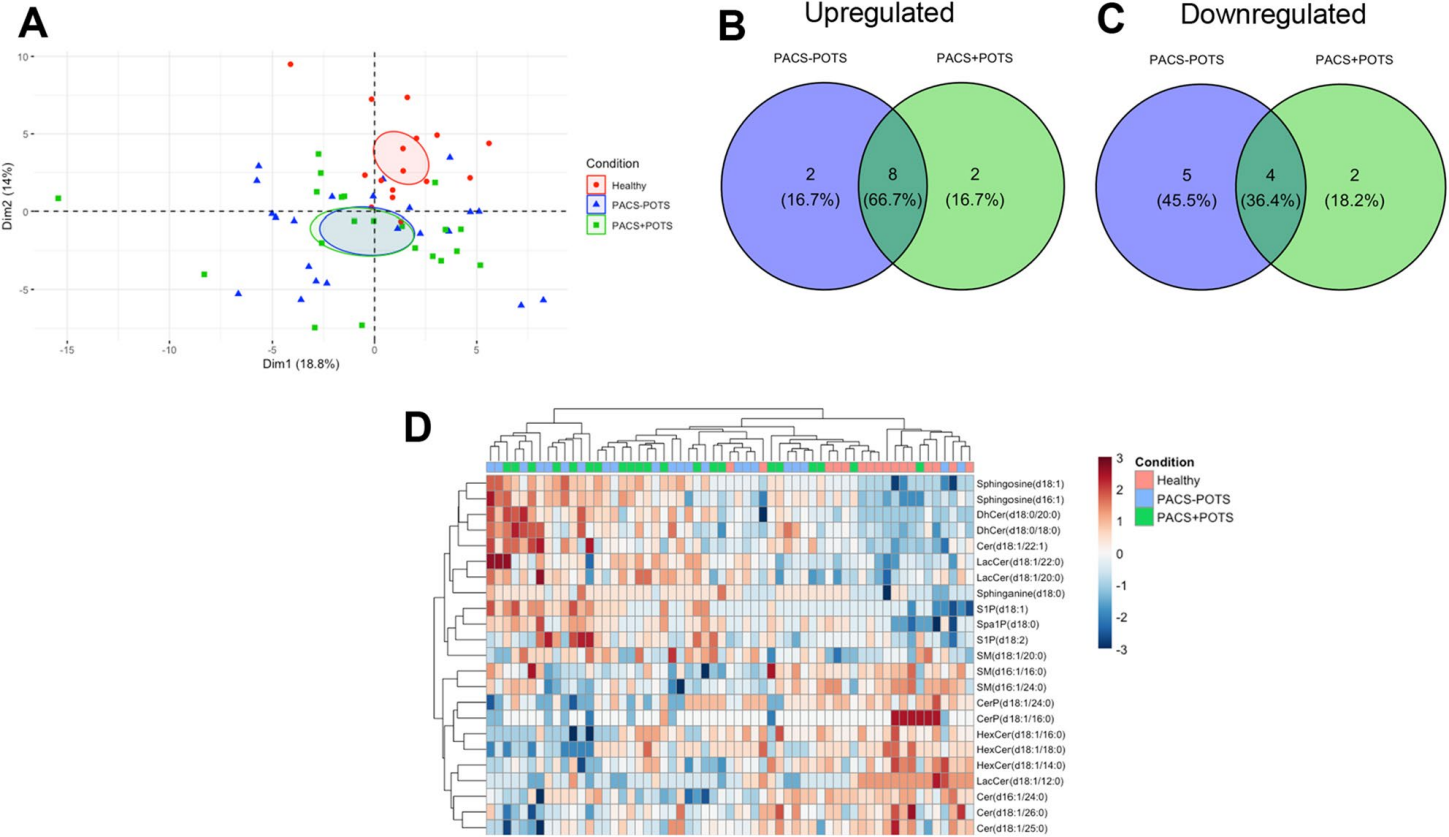
Principal component analysis (PCA) plot colored by sample group for sphingolipids (**A**). Venn diagrams of differentially upregulated (**B**) and downregulated (**C**) sphingolipids for PACS + POTS and PACS-POTS patients when compared to healthy controls. Interactive heatmap with unsupervised hierarchical clustering of samples based on sphingolipids with an adjusted p-value < 0.05 (**D**).

Again, we analyzed the data to identify differentially expressed lipids between the groups. Compared to healthy controls, we found 16 and 19 dysregulated lipids out of 88 in the PACS + POTS and PACS-POTS groups, respectively (Fig. 8A,B). Moreover, no significant differences were detected between PACS-POTS and PACS + POTS groups (Fig. 8C). A few of the most upregulated sphingolipids in both PACS groups are sphingosine (d18:1) and sphingosine (d16:1) which are the basis of sphingolipids, and sphingosine 1-phosphate (S1P) with diverse bioactive actions including critical roles in the immune system, blood pressure and endothelial function^19,20^. Interestingly, PCA analysis once again showed a clear cluster between healthy controls and PACS patients but failed to differentiate PACS-POTS from PACS + POTS group, further highlighting the similarities in plasma composition in both PACS-POTS and PACS + POTS groups (Fig. 9A). There were only two unique significantly upregulated lipids in both groups, as well as 2 and 5 unique significantly downregulated lipids in PACS + POTS and PACS-POTS groups, respectively (Fig. 9B,C). Similar to the plasma proteins and cytokines, unsupervised hierarchical clustering resulted in a clear cluster of healthy controls, but no major clusters among PACS-POTS and PACS + POTS patients (Fig. 9D). All sphingolipids with associated log2-fold changes, raw p-values and adjusted p-values and the uniquely dysregulated sphingolipids are listed in Supplementary File 3. None of the sphingolipids fell below the LOD.

Collectively, our data show clear dysregulation of the molecular plasma profile in PACS patients. This is most pronounced in cardiometabolic proteins but also in cytokines and sphingolipids compared to healthy controls (Fig. 1). Interestingly, all analyses showed a similar picture with absence of major differences between PACS patients with and without POTS. The common denominator for the outcomes of all molecular analysis is the up-regulation of pro-inflammatory proteins/molecules of importance for maintaining hemostasis, metabolism, clot formation and vascular function.

## Discussion

In the current study, we aimed at providing a molecular multi-omic characterization of cardiometabolic plasma proteins, cytokines/chemokines, and sphingolipids in PACS patients with and without POTS. This is, to the best of our knowledge, the first broad molecular characterization of PACS patients with a distinct POTS phenotype. We identified ~ 200 proteins, 11 cytokines and 16–19 sphingolipids with altered levels in previously healthy non-hospitalized PACS-patients up to 18 months after contracting a relatively mild-to-moderate SARS-CoV-2 infection. Interestingly, although major differences were detected across all analyses between healthy controls and PACS patients, the presence of POTS did not impact any of the molecular alterations seen in the group as a whole and did not differ in major aspects from the absence of POTS. Contrary to our hypothesis, this suggests that PACS is associated with distinct molecular changes regardless of POTS occurrence. Therefore, the presence of POTS in PACS may not be related with dysregulation of plasma proteins, cytokines, or sphingolipids, at least not at the discrimination level used in the current study.

It is well-known that COVID-19 infection triggers a hyperinflammatory response during the acute phase of the infection. The majority of COVIDomics studies on patients with acute COVID-19 have shown that a pro-inflammatory and pro-coagulative signature is dominant and probably drives cardiovascular perturbations and complications during ongoing illness^21^. These observations are extended in the current study which suggests that patients with PACS exhibit persistent and prolonged immune activation and dysregulation of coagulation pathways that may contribute to symptom perseverance in PACS, even 18 months after a mild acute infection. This is further corroborated by the strong dysregulation of cytokines seen in PACS patients, with important chemokines such as CCL8, CCL13^22^ and CXCL11^23^ being upregulated, showing an overall activation of the inflammatory response.

Our results suggest that alterations in hemostasis and apoptosis might play a key role in the PACS pathophysiology as several proteins involved in platelet activation and coagulation were dysregulated in PACS. This supports previous observations of COVID-19 convalescents who demonstrated microvascular endothelial dysfunction^7,24^ associated with a hypercoagulable state including platelet activation and micro-clotting^25,26^. Interestingly, previous studies have also shown dysregulation of proteins involved in platelet activation and coagulation in POTS patients without PACS^27^ highlighting the importance of hemostasis in POTS. Whether platelet activation and increased thrombogenicity induced by microvascular endothelial dysfunction constitute an amendable pathophysiological mechanism should be explored in future studies.

Interestingly, the cytokine profiling also revealed upregulation of several pro-proliferatory and pro-angiogenetic factors such as VEGF-A, EGF and TNFSF12^28–30^. Some of the previously mentioned chemokines like CCL8 have also been shown to promote angiogenesis^31^. Our proteomics dataset showed clear upregulation of PDGF-A in PACS, another potential player in angiogenesis^32^. This, together with the concurrent increases in apoptotic and hemostatic proteins, could signal that PACS patients suffer from a hypoxic environment caused by micro clots, leading to apoptosis which in turn upregulates angiogenesis to alleviate the consequences of hypoxia. This seemingly strong upregulation of angiogenic signaling is one of many potential aspects of PACS that needs to be further evaluated.

The bioactive sphingolipids, ceramides, are known to act as secondary messengers and mediate important cellular and molecular signaling. It has been shown that several ceramides predict adverse cardiovascular events with high accuracy^33^ possibly through their diverse regulatory effects on vascular reactivity^18^. More specifically, S1P, which was upregulated in our cohort, is associated with elevated blood pressure and pro-inflammatory biomarkers^19^. As they accumulate in the atherosclerotic plaque, ceramides have been implicated in the onset of lipoprotein aggregation and inflammation^34^. However, four sphingolipids implicated in cardiovascular disease as predictors of cardiovascular death in a population with coronary artery disease, namely: Cer(d18:1/16:0), Cer(d18:1/18:0), Cer(d18:1/24:1) and Cer(d18:1/24:0) were not significantly dysregulated in the groups compared^35^. This might suggest that these ceramides are implicated in plaque vulnerability in patients with established atherosclerotic plaques. However, the subjects included in the current study do not have known or at risk for coronary artery disease, which might explain the lack of dysregulation of these certain ceramides. Beyond the cardiovascular system, sphingolipids have been implicated in the cellular processes of skeletal muscle cells^36^. Hence, the observation of persistent alterations of sphingolipids in PACS may not only suggest a predisposition to cardiovascular disease but also to chronic fatigue among PACS patients. However, the potential impact of these alterations on plaque formation and muscle fatigue remains to be established in larger cohorts with longer follow-up.

We found very small differences in protein, cytokine and sphingolipid dysregulation between PACS patients with and without POTS. This suggests that POTS may exist on the background of biochemical alterations related primarily to PACS. What specific mechanisms cause development of cardiovascular autonomic dysfunction in general, and POTS in particular, on top of PACS, could not be established. We speculate that since acute COVID-19 is known to trigger a profound autoimmune response^37,38^ and there is evidence of autoimmunity being a potential disease mechanism in classical POTS^39,40^, autoimmune factors may be decisive for POTS development in PACS.

Collectively, our findings from a large proteomic analysis, cytokine profiling and sphingolipid quantification suggest major alterations of importance in mediating pro-inflammatory signaling in PACS. Future studies should confirm the findings in larger cohorts, assess the prognostic value of the observed changes, investigate the association of the dysregulated molecules to clinical symptoms and most importantly investigate the potential therapeutic targets aiming at restoring the observed changes. The current study suggests that such targets might include anti-inflammatory therapies, immunotherapies, and anticoagulants. Indeed, drugs targeting inflammation, the immune system and the coagulation cascade with promising results have been performed or are currently under investigation (NCT: 05823896)^41,42^. The overlap in COVIDomics^21^ between the acute infection and PACS not only suggest a prolonged activation of a pro-inflammatory, pro-coagulative and immune activation state, but also that beneficial pharmacological targets in the acute infection might also alleviate and reduce the symptom burden in PACS.

## Limitations

This study has several limitations. The results of the study are based on associations between molecular dysregulation and clinical phenotypes, and any causative relationship should be interpreted with great caution. The limited sample size does not allow for in depth analysis of associations of molecular dysregulations and clinical symptoms. Moreover, the analyses in this study were targeted and thereby limited, which means that we may have missed several important plasma proteins, cytokines, and lipids. Furthermore, we cannot rule out that protein, cytokine, and sphingolipid dysregulation also exist long-term after SARS-CoV-2 infection and vaccination also in asymptomatic patients i.e. in individuals who did not develop PACS. Hence, these results should be verified using a control group that have had SARS-CoV-2 infection with a complete recovery. An additional limitation is that PACS patients in this study contracted SARS-CoV-2 infection during the first and second pandemic wave. The virus has since mutated, mass vaccination has been performed in many countries and anti-viral treatment is indicated in some patients with risk factors for severe COVID-19. Therefore, the results from this study may not be directly extrapolated to later cases of SARS-CoV-2 infections and in vaccinated patients or patients with anti-viral treatment during the acute phase of COVID-19. However, including patients from the first and second wave offer a unique opportunity to document the natural progression of PACS, which represents a strength of this study. The study was almost exclusively performed on female patients, hence extrapolation to male patients should be made cautiously. For unknown reasons, females contracting COVID-19 are more likely to suffer from PACS^43^. Finally, the study is limited in size without validation of the dysregulated proteins, cytokines, and sphingolipids by other methods.

## Conclusions

Patients with PACS demonstrate distinctly altered plasma protein, cytokine and lipid profiles compared with healthy controls. In particular, alterations in hemostasis, T-cell proliferation, apoptosis and amino acid metabolism may be potentially involved in symptom development and maintenance in PACS. Interestingly, plasma profile of PACS patients with and without POTS do not substantially differ, implying that PACS is associated with alterations in plasma profile regardless of POTS presence. Other pathophysiological mechanisms may be responsible for the development of POTS in PACS. Collectively our data suggest that the pro-inflammatory/proliferative and pro-coagulative state in PACS represents an important phenotype in the pathophysiology of the disease and might largely explain the disease burden of the population. This may lay the foundation for future clinical investigations with focus on inflammation resolution, resolution of micro clots and rearrange the hemostasis to alleviate the disease burden or dissect possible biomarkers for disease progression in PACS.

### Supplementary Information

Supplementary Information 1.

Supplementary Information 2.

Supplementary Information 3.

Supplementary Information 4.

Supplementary Information 5.

## Data Availability

The authors confirm that the data supporting the findings of this study are available within the article and its supplementary materials (raw data in Supplementary File 5).

## Abbreviations

LC–MS: Liquid chromatography-tandem mass spectrometry
LOD: Limit of detection
MAPS: Malmö postural orthostatic tachycardia syndrome
PACS: Post-acute sequelae of COVID-19
PCA: Principal component analysis
POTS: Postural orthostatic tachycardia syndrome
